# Galectins in the Pathogenesis of Common Retinal Disease

**DOI:** 10.3389/fphar.2021.687495

**Published:** 2021-05-17

**Authors:** Bruna Caridi, Dilyana Doncheva, Sobha Sivaprasad, Patric Turowski

**Affiliations:** ^1^ UCL Institute of Ophthalmology, University College London, London, United Kingdom; ^2^ NIHR Biomedical Research Centre, Moorfields Eye Hospital NHS Foundation Trust, London, United Kingdom

**Keywords:** retina, diabetic retinopathy, age-related macula degeneration, VEGF, angiogenesis, leakage

## Abstract

Diseases of the retina are major causes of visual impairment and blindness in developed countries and, due to an ageing population, their prevalence is continually rising. The lack of effective therapies and the limitations of those currently in use highlight the importance of continued research into the pathogenesis of these diseases. Vascular endothelial growth factor (VEGF) plays a major role in driving vascular dysfunction in retinal disease and has therefore become a key therapeutic target. Recent evidence also points to a potentially similarly important role of galectins, a family of β-galactoside-binding proteins. Indeed, they have been implicated in regulating fundamental processes, including vascular hyperpermeability, angiogenesis, neuroinflammation, and oxidative stress, all of which also play a prominent role in retinopathies. Here, we review direct evidence for pathological roles of galectins in retinal disease. In addition, we extrapolate potential roles of galectins in the retina from evidence in cancer, immune and neuro-biology. We conclude that there is value in increasing understanding of galectin function in retinal biology, in particular in the context of the retinal vasculature and microglia. With greater insight, recent clinical developments of galectin-targeting drugs could potentially also be of benefit to the clinical management of many blinding diseases.

## Introduction

The retina is a sensory tissue of vertebrate and cephalopod eyes harbouring photoreceptor cells and their connecting neurons and feeding visual information to the brain. In humans, the retina lines the inner part of the posterior two-thirds of the eyeball. Diseases and traumas of the retina are collectively referred to as retinopathies. Often, the term retinopathy is used exclusively to describe diseases, which affect the retinal microvasculature. Here, we also include traumatic disease of the retina and age-related macular degeneration (AMD), since their pathologies are also likely to involve galectins (Table 1). Retinopathies often progress to blindness and thus are very debilitating for patients. Diabetic retinopathy (DR) and AMD alone account for at least 50% of registered blindness worldwide and conservative estimates suggest that, worldwide, 170 million have AMD, and at least 30% of people with diabetes have DR (Pennington and DeAngelis 2016; Yau et al., 2012). With increasing prevalence of underlying systemic diseases and environmental stressors, these numbers will only increase and put additional economic strain on health and social services.

**TABLE 1:** Clinical and biological features of retinal diseases and confirmed galectin involvement. See main text for additional information.

Diseases of the retina		Clinical features	Biological features	Galectins involved
Physical damage	Retinal tear	Floaters; Photopsia; Vitreous Haemorrhage; Can result in retinal detachment Landau and Kurz-Levin (2011)	Vitreous liquifies with age and eventually detaches from the retina Landau and Kurz-Levi (2011)	—
	Retinal detachment	Light flashes, Floaters; Photopsia; Blurred vision; Reduced peripheral vision Landau and Kurz-Levin, (2011)	Rhegmatogenous (most common): Caused by hole in the retina.	Gal-3 accumulation in subretinal fluid of patients with rhegmatogenous retinal detachment Poulsen et al. (2020)
			Tractional (e.g. poorly controlled diabetes): Caused by traction from scar tissue on the retinal surface	
			Exudative: Not caused by holes or tears, but often by tumours or inflammation Landau and Kurz-Levin (2011); Steelman and Li (2014)	
	Macular hole	Blurring; Distortion	Full-thickness defect of retina involving fovea Ittarat et al. (2020)	—
	Proliferative Vitreoretinopathy (PVR)	Blinding complication of fibrovascular proliferation	Proliferative and inflammatory response of a variety of retinal cells–RPE undergo EMT Alge et al. (2006); Priglinger et al. (2016)	Gal-1 and Gal-3 reduce RPE cell adhesion and spreading Alge et al. (2006); Priglinger et al. (2016)
	Epiretinal membrane	Metamorphopsias and central vision impairment	Abnormal growth of tissues on the retinal surface Wang et al. (2016)	No direct evidence for galectin, but key role of Gal-3 in fibrosis Slack et al., (2021)
Complex, multifactorial	Diabetic Retinopathy (DR)	Microaneurysms; Intraretinal haemorrhages; Cotton-wool spots; Venous beading; Vascular loops	Degeneration and loss of pericytes; Proliferation of endothelial cells and thickening of the basement membrane; Capillary occlusion and reduced capillary flow; Inflammation; Increased platelet stickiness and aggregation; Increased production of angiogenic factors, especially VEGF Antonetti et al., (2021).	Gal-1 upregulated in the vitreous and aqueous humour of PDR patients Abu El-Asrar and Ahmed, (2020);Kanda et al., (2017); Ridano et al., (2017)
				Gal-1 is upregulated in retinal tissue of mice with features of DR Kanda et al., (2017)
				Gal-1 upregulated in neovascular tufts of OIR mice Liu et al., (2009)
				Gal-3 KO mice showed less retinal disease Canning et al. (2007)
	Retinal vein occlusion (RVO)	Blocked central or branch retinal vein causing widespread retinal haemorrhages and macular oedema (Blair and Czyz (2020))	Neovascular complications	Vascular complications similar to DR/DMO, indicating Gal-1 involvement (see main text)
				Gal-3 may protect retina as it does in ischemic stroke Wesley et al. (2020)
	Retinopathy of prematurity (ROP)	Late stages may present with leucocoria (white reflex); Nystagmus with abnormal eye movements; Bilateral retinal detachment; Falciform fold and pthisis bulbi Dogra et al. (2017)	Delayed retinal vascular development due to hyperoxia and low serum IGF1 in premature babies; Reflex vasoconstriction; Pathologic angiogenesis; High VEGF in the vitreous Margalit and Srinivas (2003)	Galectins not directly involved, but ROP management uses anti-VEGFs, suggesting roles for galectins (*see* main text)
	Hypertensive Retinopathy (HR)	Mild or vasoconstrictive (silver or copper wiring)	Mild–retinal arterial narrowing of the vessels or sclerosis; moderate–additional intimal thickening and arterial narrowing; focal or diffuse arterial wall opacification Malignant–optic nerve swelling Harjasouliha et al. (2017); Kabedi et al. (2014); Tsukikawa and Stacey (2020)	—
		Moderate or sclerotic phase (hemorrhages, microaneurysms, cotton-wool spots, exudates)		
		Malignant or exudative phase (moderate retinopathy and optic disk swelling)		
	Age-related macular degeneration (AMD)	Dry (non-neovascular)–slow but progressive decrease in visual acuity, increasing light sensitivity, and reading difficulties	Dry–yellow lesions (drusen) below the RPE, atrophy or hyperpigmentation of the RPE	Gal-1 upregulated in a model of wet AMD Wu et al. (2019)
		Wet (neovascular)–sudden, often quite marked, decrease in visual acuity; can results in permanent reduction of vision as well as a central scotoma	Wet–neovascular growth of the choroid; bleeding and exudation from these vessels can damage the outer retina, leading to photoreceptor degeneration Margalit and Srinivas (2003); Landau and Kurz-Levin (2011)	Gal-2, -7, -8 upregulated in RPE/choroid samples of some forms AMD; Gal-8, -12 downregulated in neuroretina of pre-AMD patients, and Gal-3 upregulated in most forms of AMD Newman et al. (2012)
				Gal-3 upregulated in choroid samples from advanced dry AMD Yuan et al. (2010)
Inherited retinal diseases	Retinitis Pigmentosa (RP)	Signs include optic nerve pallor, constricted retinal vessels, and bone spicule pigmentation in the periphery	Progressive loss of retinal rod photoreceptor cells followed by subsequent degeneration of cones→ increased reduction of retinal function and eventually retinal atrophy Hartong et al. (2006);Landau and Kurz-Levin (2011); Margalit and Srinivas (2003).	Gal-3 expression elevated in Müller cells in mouse model of RP Roesch et al. (2012)

Whilst the pathogeneses of the various retinopathies is usually complex and still under intense and continuous investigation, they all feature one or a combination of: vascular dysfunction (culminating in vessel leakage and microbleeds), angiogenesis, inflammation, and oxidative stress (Campochiaro 2015). Intuitively, this suggests critical involvement of galectin family members, with their demonstrated roles in these or similar processes in the context of other pathologies such as cancer, fibrosis and heart disease, to name just a few (Johannes et al., 2018). This review aims at presenting accumulating direct, as well as circumstantial, evidence for critical roles of these specialised carbohydrate-binding proteins in the pathogenesis of retinopathies. In many cases, circumstantial and hypothetical evidence is strong but calls for targeted investigation, and we will highlight promising routes of future research. Lastly, in light of their druggability, we will evaluate if therapeutic targeting of galectins holds promise in the clinical management and treatment of retinopathies.

## Galectins and Their Biology

**FIGURE 1:**
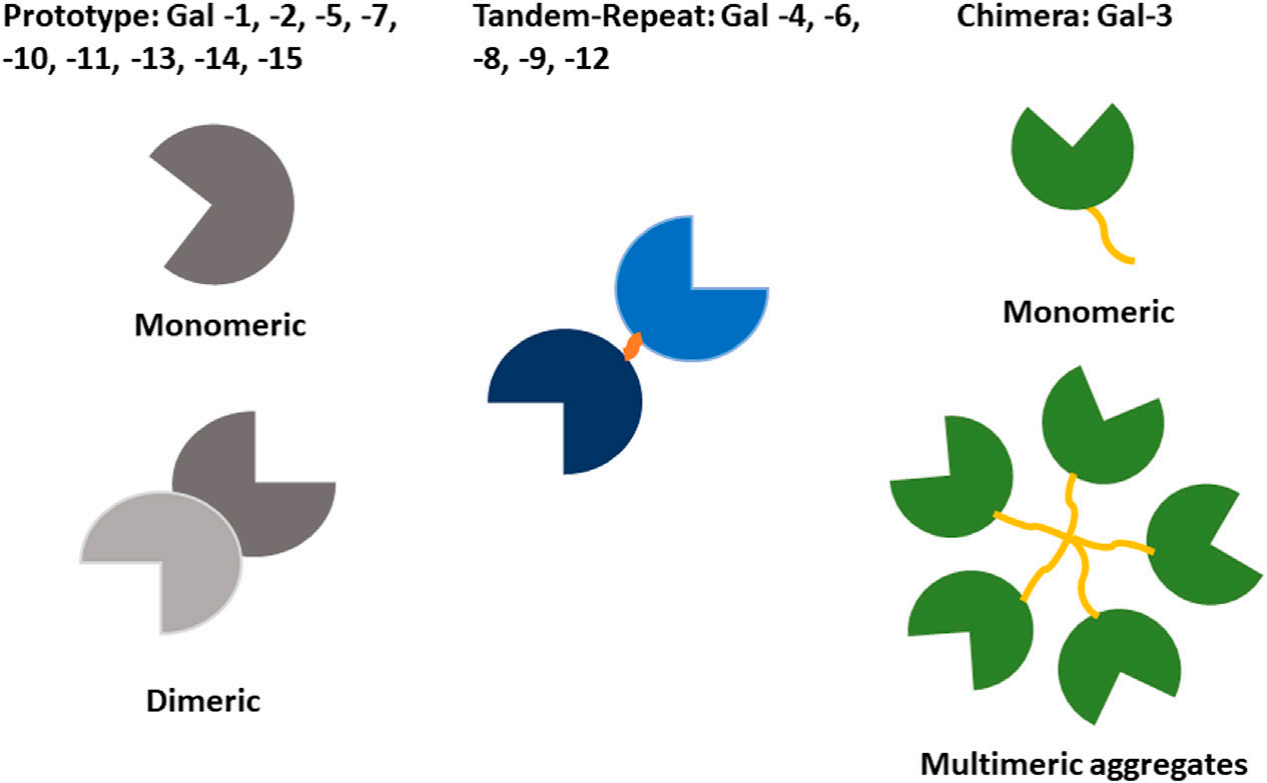
Classification of galectin proteins. Functionally, galectins always have at least two CRDs, either located within the same polypeptide or by multimerisation. Three galectin subtypes can be distinguished based on the structural organization of the conserved carbohydrate recognition domain (CRD). Prototypic galectins contain a single CRD forming homodimers. Tandem repeat galectins contain two distinct CRDs. Chimeric galectins contain a single CRD and can form multimers (only Gal-3 belongs to this group).

Galectins comprise a family of animal lectins defined by the presence of a highly conserved carbohydrate recognition domain (CRD) with specificity for β-galactose-containing oligosaccharides. Galectins are devoid of folded domains other than CRDs. The typical CRD consists of ca. 135 amino acids compactly folded into a sandwich structure of two 5- (or 6-) stranded β-sheets. Galectins are encoded by “lectin, galactoside-binding, soluble” (LGALS) genes, with 15 distinct genes identified in mammals (Rabinovich, et al., 2002; Rabinovich 1999). Using structural criteria, Hirabayashi and Kasai have categorised galectins into proto-type, tandem-repeat type, and chimera type (Figure 1; Kasai and Hirabayashi, 1996). Prototype galectins contain a single CRD, form non-covalent homodimers and include galectin-1 (Gal-1), -2, -5, -7, -10, -13, -14, and -15. By contrast, Gal-4, -6, -8, -9, and -12 are tandem-repeat galectins, which have two distinct CRDs in the same polypeptide. Gal-3 is the only chimera-type galectin in vertebrates. It has one CRD at its carboxyl terminus, which is preceded by a long proline-, glycine-, and tyrosine-rich N-terminal non-lectin domain. Gal-3 exists as a monomer but also self-assembles into multimers (up to pentamers).

Galectins are present both intracellularly (within membrane compartments, cytosol, and nucleus) and extracellularly. Notably, they are synthesised as cytosolic proteins and, when secreted, utilise a non-canonical pathway not implicating the endoplasmic reticulum or Golgi apparatus. Whilst the exact molecular details of galectin secretion are not fully understood, their unconventional secretory pathway appears to be also used by interleukin (IL)-1β, annexin A1 and fibroblast growth factor, with experimental evidence for direct translocation across the plasma membrane, as well as processes driven by post-Golgi compartments such as endosomes or exosomes, but also plasma membrane microvesicles (Popa, et al., 2018).

However, the question of secretion of galectins should not detract from the fact that these lectins are predominantly found in the cytosol and nucleus, where they are stored but also perform important functions. Intracellular functions of galectins are wide-ranging and comprise intracellular signalling, cytoskeletal and endosomal organisation and nuclear RNA splicing (Johannes et al., 2018). Galectins also play prominent roles in endosomal sorting and the autophagy of endocytic vesicles or compartments. Within the extracellular compartment, galectins display the capability to bind to and, by virtue of their bi- or multivalency, cross-link cell surface receptors resulting in activation and modulation of a broad range of signalling pathways regulating cellular processes as diverse as cell growth, cell-cell adhesion, autoimmune responses, inflammatory and apoptosis (Johannes et al., 2018).

Galectins bind both carbohydrate- and non-carbohydrate-containing ligands via their CRD, with the latter naturally more widespread in the cyto- and nucleosol. Nevertheless, galactose-binding is central to galectin biology, in particular that linked to extracellular receptor activation. Indeed, CRDs bind galactose with low affinity and N-acetyl lactosamine (Galβ1, 3GlcNAc or Galβ1, 4GlcNAc), a common disaccharide present on many N- or O-linked glycans, with micromolar affinity (Elola et al., 2005). In turn, increased binding affinity in the submicromolar range is seen toward polylactosamines (lactosamine complex of repeating chains) or lactosamine presented in the structural context of a glycoprotein (Cho and Cummings, 1995). In this regard, structural analysis of CRD of different galectins shows slight but important variations in carbohydrate-binding specificities, thus explaining their ability to specifically modulate distinct glycolipid and glycoprotein receptors with highly varied biological outcomes (Brewer, 2002).

Of the 15 members of the galectin family, only Gal-1, -3, -4, -8 and -9 have been found significantly expressed in the nervous system (De Jong, et al., 2020; Siew and Chern 2018). Gal-1 expression was mostly observed in GFAP-positive astrocytes and Müller glia, whereas Gal-3 expression was observed mostly in Iba1-as well as CD16/32-positive cells, the inflammatory cells of the nervous system (Bonsack and Sukumari-Ramesh 2019; Hirose, et al., 2019). Vascular endothelial cells express several galectin family members, including Gal-1 and Gal-3 (Thijssen, et al., 2007).

RNAseq analysis of C57BL/6 mouse retinae shows that only transcripts for LGALS1, 2, 8 and 9 are expressed in healthy tissue, with LGALS1, 2 and 9 restricted to the endothelium. During inflammation, induced by experimental autoimmune uveitis (EAU), LGALS1 and 9 are also upregulated in the neuroretina and LGALS3 in the endothelium, whilst endothelial LGALS2 expression is completely suppressed (Lipski, et al., 2020). Other pre-clinical and clinical expression studies comparing healthy and pathological retinae are discussed within relevant sections below, but collectively they show that galectins are abundantly expressed in the retina, in particular Gal-1, -3, -7, -8, -9, with changes in their expression in human retinal disease or rodent disease models also well documented (Bogdanov, et al., 2014; Newman, et al., 2012).

## Diseases of the Retina and Galectins

### Retinal Topology, Structure and Function

**FIGURE 2:**
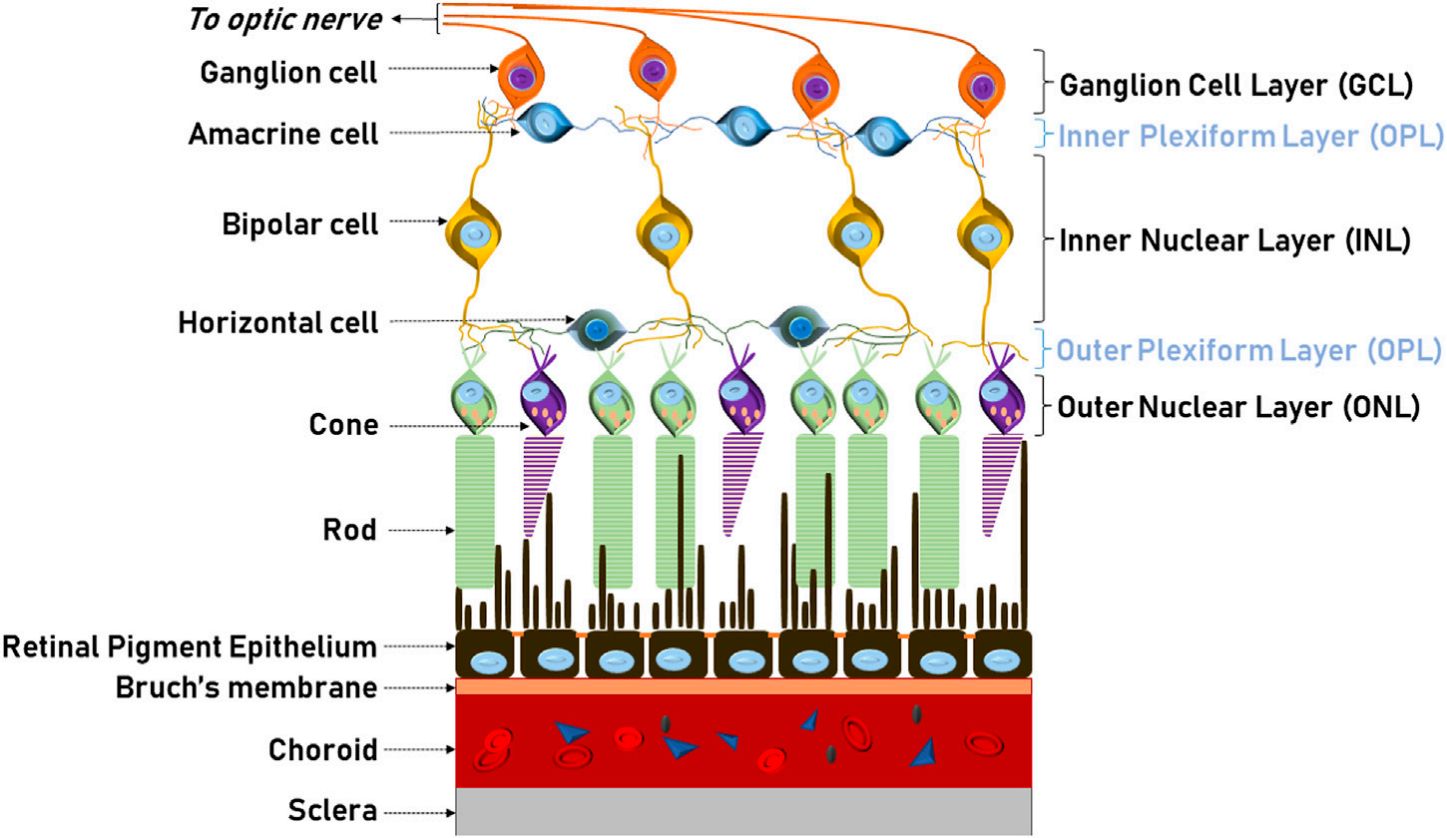
Structure and morphology of the retina. Schematic illustration of the neural circuit of the retina showing the five neuronal cell types: photoreceptors, horizontal, bipolar, amacrine and ganglion and supporting cells. Photoreceptor outer segments (cones and rods) are apically associated and supported by the RPE. The blood supply to the outer retina is from the choroid situated between Brunch’s membrane and the sclera.

**FIGURE 3:**
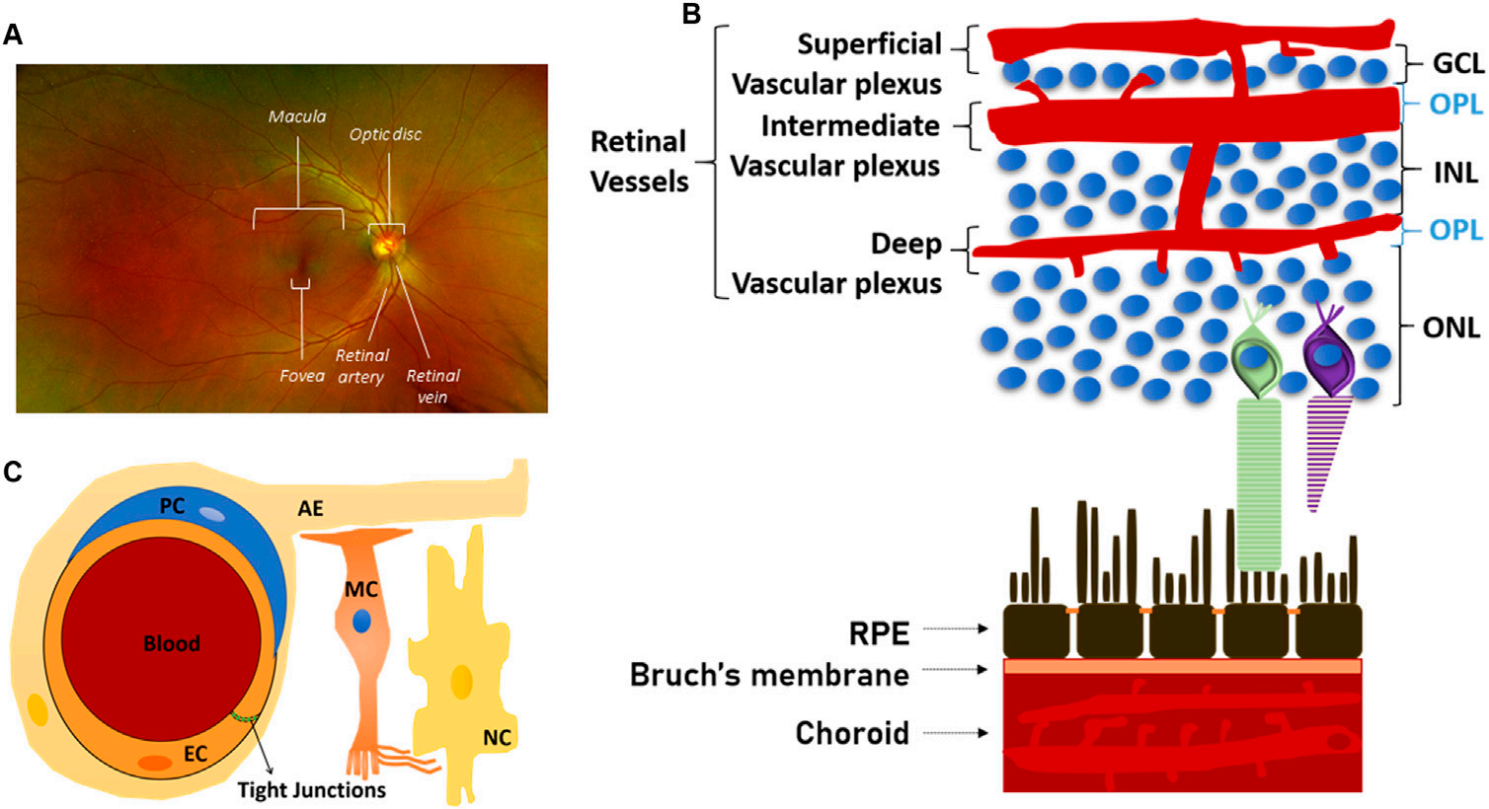
Structure and morphology of the retinal vasculature **(A)** Full colour retinal fundus image of a normal human eye. The optic disc resides in the middle of the retina where major blood vessels branch throughout the eye except one area–the fovea, which is situated in the centre of the macula **(B)** Structural organisation of the retinal vasculature. Schematic illustration of the three (superficial, intermediate and deep) main layers of the retinal vasculature. The photoreceptor layer is completely avascular **(C)** Schematic representation, at the microvascular (capillaries) level, of the neurovascular unit, which is formed by endothelial cells (EC), pericytes (PC), astrocytes endfeet (AE), Müller cells (MC), which also interact with all retinal neuronal cells (NC) as illustrated in Figure 2.

The retina is an extension of the CNS, with diverse neuronal cells acting in concert to absorb light stimuli, translate these into modulated and integrated electrical and chemical signals before they are transmitted via the optical nerve to the visual cortex (Kolb, 2005) (Box 1; Figures 2, 3). Photoreceptor cones and rods undergo daily renewal via a continuous cycle of resynthesis and shedding of outer segments. Shed receptor components are taken up by the retinal pigment epithelium (RPE). RPE are among the most metabolically active cell types, and their primary function consists of disposing retinal waste products and recycling photoreceptor components (e.g. renewing photopigment) (Strauss, 2005). Other crucial functions of the RPE are: epithelial transport of nutrients, ions and water; endocrine secretion; immune regulation; and the absorption of scattered light. RPE also create an important cellular barrier between the neural retina and the choroidal blood circulation (see below). Unsurprisingly, RPE integrity and health is instrumental to overall retinal health and RPE dysfunction is associated with many of the retinal diseases described below (Caceres and Rodriguez-Boulan, 2020; Lakkaraju et al., 2020).

Box 1 **Structure and function of the vertebrate retina.**

**FIGURE 4:**
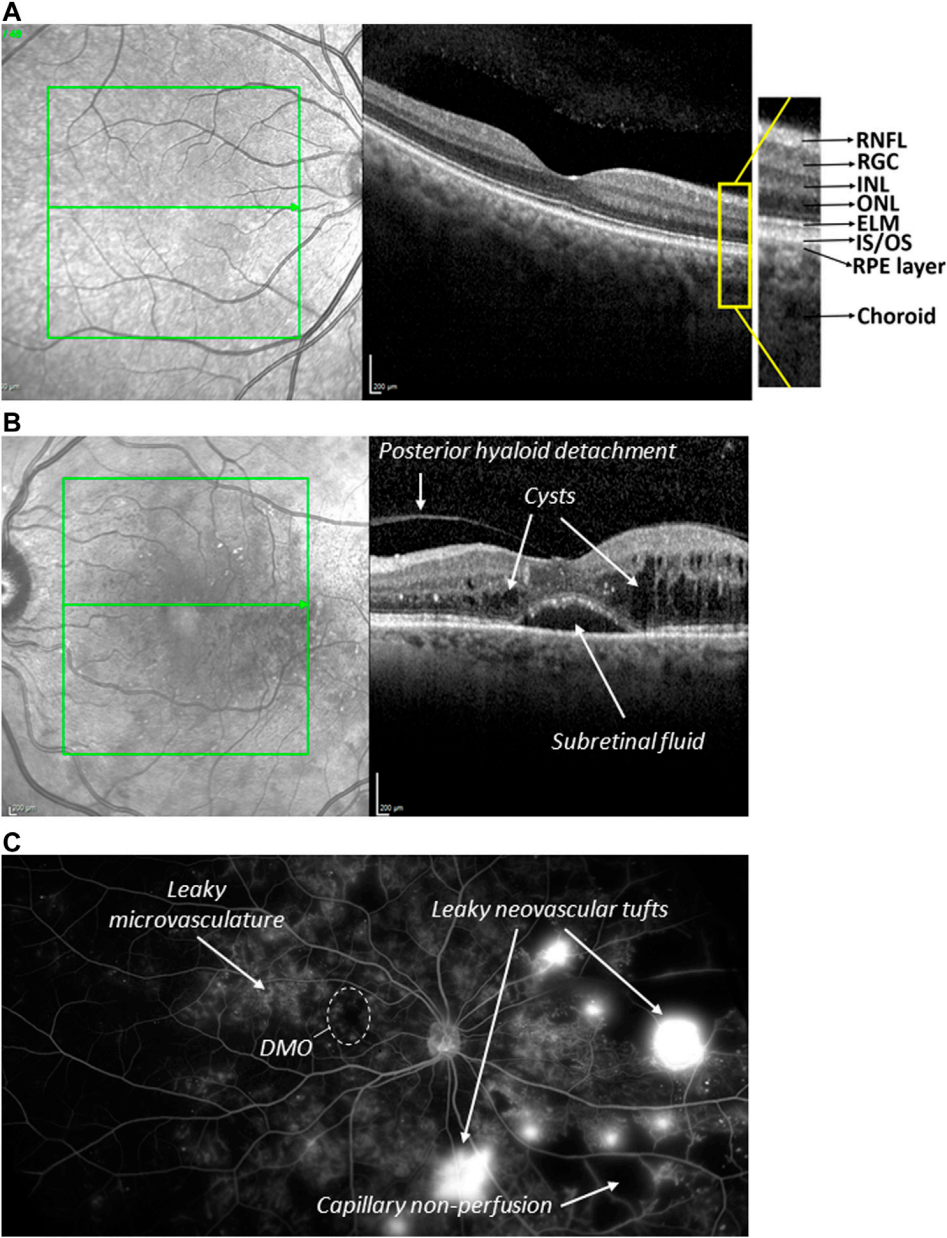
Normal and diseased human retina **(A)** Optical coherence tomography (OCT) image sectioning the macular area of a healthy retina (right). The corresponding fundus image of the macular area (green box) is shown on the left, in which the section of tomographic scan indicated by the arrow. The fovea depression is seen in the centre. RNFL = retinal nerve fibre layer. RGC = retinal ganglionic cells. INL = inner nuclear layer. ONL = outer nuclear layer. ELM = external limiting membrane. IS/OS = inner segment/outer segments. **(B)** OCT image of a macula of a patient with DMO. Severe oedema results in fluid filled cysts around the fovea, subretinal fluid just underneath the fovea, and posterior hyaloid detachment. Retinal layering and fovea depression are lost **(C)** Wide field angiography image of a PDR eye. Clearly visible are observed abnormal growth of blood vessels on the optic nerve, neovascularization (new blood vessels), microaneurysm and capillary non-perfusion.

In all vertebrates, the retina comprises five neuronal cell types: photoreceptors, bipolar, ganglion, horizontal and amacrine cells (Figure 2). Their cell bodies are located in the inner, outer nuclear and ganglion cell layers, with all connecting synapses located in the inner and outer plexiform layers. Photoreceptors contain opsins, polyene chromophore-containing GPCRs that, in connection with highly efficient heterotrimeric GPCR–driven signal transduction, induce cell hyperpolarisation in response to light and the generation of a primary neuronal stimulus (stop to glutamate release at the primary synapse). Photoreceptors are present as either rods and cones, with differing sensitivities for light (Kolb, 2005; Hoon, et al., 2014). Rods can detect even a single photon and thus control dim-light vision. Cones are involved in bright-light, high perception vision where each cone photoreceptor displays sensitivity to a specific light wavelength (thus also generating the signals for colour vision). The primary synaptic signals from rods and cones travel via bipolar to ganglion cells. Along the way, signals are integrated and modulated (e.g. adapted to light conditions) by horizontal cells (interconnecting multiple photoreceptors) and amacrine cells (the interneurons of the retina) and then transmitted directly to the brain by the ganglion cell axons (Kolb, 2005; Hoon et al., 2014; Masland, 2001).
In humans, the retina is a circular tissue of about 3–4 cm, which notably does not display the same light sensitivity throughout (Figure 3A; Masland, 2001). At the center of the retina, the optic disc is a blind spot, which is devoid of rod and cones, and where ganglion nerves gather to connect to the brain through the optic nerve. This is also the area where the central retinal artery enters the retina and from which the major radial blood vessel emanates. Next to the optic disc is the central area of the retina (with all other retinal sectors considered peripheral). This ca. 6 mm wide circular field is responsible for high-resolution, colour vision and known as the macula. At its centre sits the fovea, a ca. 1.5 mm wide depressed pit, which contains only cones (Figure 4A). The centre of the fovea is entirely blood-vessel free to allow for completely unimpeded light sensing. Around half of all nerve fibres entering the optic disc transmit information generated at the fovea, illustrating the key role of the fovea for high-acuity vision (Bringmann et al., 2018).

The brain and the retina have disproportionally high metabolic demands but notably lack an energetic reservoir. Hence, they have evolutionarily developed sophisticated vascular beds, which judiciously regulate the supply of nutrients and the disposal of waste (Wong-Riley, 2010). Focusing on the retina, the central retinal artery and the choriocapillaris are the main vessel systems that supply blood to the inner retina and the RPE and the outer retina, respectively (Figure 3B; Joyal, et al., 2018). Branching out from the central retinal artery, three capillary layers then supply blood to the inner retina: the superficial, intermediate and deep retinal vasculature (Gariano and Gardner, 2005). Notably, despite having the highest demand for oxygen, this architecture leaves the photoreceptor layer as completely avascular. Thus, photoreceptors primarily rely on the choriocapillaris and the activity of the RPE to provide much of the required oxygen and nutrients. In this context, it has been proposed that this morphological uniqueness of the retina may lead to more pronounced relative hypoxia and thus render this neuronal tissue more sensitive to vascular dysfunction, as seen e.g. in diabetes (Lange and Bainbridge, 2012).

Any neuronal environment and its delicate ionic balance necessitate strong protection, and therefore neuroglia is separated from the blood circulation by endothelial or epithelial cell barriers. So called blood-retinal barriers (BRBs) operate on the level of the retinal microvasculature (inner BRB) and the RPE (outer BRB) (Figure 3B; Box 2) (Biswas et al., 2020; Caceres and Rodriguez-Boulan, 2020; Simó et al., 2018). Notably, the inner BRB endothelial cells should always be considered in the context of their surrounding abutting and regulating cells, in particular pericytes, astrocytes and Müller cells, which are collectively referred to as a neurovascular unit (Figure 3C). Importantly, most retinopathies are linked to some dysfunction of the neurovascular unit or the RPE, with a breakdown of at least one of the BRBs contributing significantly to pathogenesis.

Box 2 **Blood-neural barriers in the retina.**

In analogy and morphological similarity to blood-brain barriers, the retina has sophisticated blood-retinal barriers (BRBs) (Figure 3B; Biswas et al., 2020; Simó et al., 2018). Specifically, the inner BRB operates on the level of the retinal microvasculature, whereas the outer BRB is constituted by the RPE (Caceres and Rodriguez-Boulan, 2020). Blood vessels of the inner BRB are highly similar to those found in the brain, with endothelial cells featuring highly impermeable paracellular tight junctions, a notable absence of pinocytic vesicles and a high expression of membrane efflux pumps of the ABC transporter family. To ensure efficient delivery of nutrients to the underlying neurons, these endothelial cells have developed compound–selective transport systems which consist of luminal and abluminal membrane transport proteins and receptor- and absorptive-mediated transcytosis. Importantly, the endothelial cells of the inner BRB should always be considered in the context of their surrounding abutting and regulating cells, in particular pericytes, astrocytes and Müller cells (in the retina), which are collectively referred to as the neurovascular unit (Figure 3C). The outer BRB is exclusively made up of the RPE, with epithelial tight junctions forming a formidable molecular barrier between the generally highly permeable choroidal vasculature and the neuroretina (Caceres and Rodriguez-Boulan, 2020). Similarly to the endothelial cells of the inner BRB, RPE also express a wide variety of membrane transporters assuring nutrient and waste disposal requirements of the retina, in particular the photoreceptors.

Retinopathies can affect any layer of the retina and will often go unnoticed unless central vision (provided by the macula; Box 1) is affected. Notably, many diseases manifest specifically within the macular area, with macular blood vessel dysfunction often a major contributing factor (Table 1).

### Physical Damage of the Retina

Physical damage of the retina is overall quite rare and can generally be corrected by vitreoretinal surgery. Injuries to the retina include retinal detachment (Qureshi and Steel, 2020), retinal tear (Ang et al., 2010) and traumatic macular hole (Ittarat, et al., 2020). Whilst retinal surgery bears no therapeutic connection to galectins, proliferative vitreoretinopathy (PVR), a relatively frequent, often blinding complication of vitrectomy, does. Indeed, PVR is a proliferative and inflammatory response of a variety of retinal cells, driven by cytokines such as tumour necrosis factor alpha (TNFα), transforming growth factor β2 and interleukins (Schaub et al., 2021). In particular, RPE undergo epithelial to mesenchymal transition (EMT), during which galectins appear to be upregulated, at least when modelled *in vitro*. Both Gal-1 and Gal-3 reduce RPE cell adhesion and spreading, induce growth receptor signalling, and may thus directly drive EMT (Alge et al., 2006; Priglinger et al., 2016). There is no established treatment of PVR but anti-inflammatory or antiproliferative approaches have been tried, without any pharmacological substance having yet emerged for either treatment or prophylaxis (Schaub et al., 2021). Given the important and wide-reaching roles both these galectins play in inflammation and aberrant proliferation, further research into a pathogenic role with therapeutic potential appears warranted (Obermann et al., 2017). Recently, it has also been reported that many proteins, including Gal-3, accumulate selectively in the subretinal fluid (the fluid accumulating between the neuroretina and the RPE) of patients with rhegmatogenous retinal detachment. However, what this observation may mean for disease progression or management needs to be clarified with supportive studies (Poulsen et al., 2020). Another retinal disease associated with the loss of the macular architecture is epiretinal membrane. Its cause is an abnormal growth of tissues on the inner retinal surface at the macular area, with fibroblasts and myofibroblasts found in overlaying fibrotic patches (Wang, et al., 2016). This indicates a potential role for Gal-3, which plays a key role in fibrosis by activation of profibrotic factors and collagen production in e.g. fibroblasts and macrophages, and has been advocated as a therapeutic target in fibrotic disease (Slack, et al., 2021).

### Diabetic Retinopathy

Conflicting hypotheses exist as to the primary cell type that drives diabetic retinopathy (DR) in response to systemic hyperglycaemia. Here we present the hallmarks of the disease from a predominantly vascular point of view. DR affects retinal blood vessels, and these blood vessel changes currently serve as a key biomarker in the diagnosis and stratification through basic or sophisticated retinal imaging.

DR is the most common microvascular problem of diabetes and the principal cause of blindness in adults 24–70 years of age; eventually, it affects over 36% of the diabetic population (Antonetti et al., 2021). DR is a direct consequence of persistent hyperglycaemia and manifests itself in microangiopathy. Pathological permeability and neovascularization are other hallmark features and may be a consequence of capillary occlusion. Depending on the absence or presence of neovascularization, DR may be classified as either nonproliferative (NPDR) or proliferative (PDR). NPDR generally constitutes an early stage of the disease with clear signs of lipid exudates, nerve fibre damage, basement membrane thickening, microaneurysms, dot and blot haemorrhages, cotton-wool spots, and capillary nonperfusion owing to microvascular damage and pericyte loss (Duh et al., 2017; Nentwich and Ulbig, 2015). NPDR patients can then progress to PDR and/or diabetic macular oedema (DMO) (Figures 4B, C; Antonetti et al., 2021). Neovascularization is driven by angiogenesis and results in retinal and vitreous haemorrhages and consequently decreased visual acuity and retinal detachment (Antonetti et al., 2021; Duh et al., 2017). The risk of progression to PDR may be reduced by tight glycaemic control during NPDR (Blighe et al., 2020). DMO is a manifestation of the disruption of the BRB in the macula, typically associated with a presence of leaked lipids (hard exudates) and a thickening of this area, often leading to a complete morphological disruption of the foveal pit and high acuity vision (Figure 4C).

Persistent exposure of the retina to hyperglycaemia induces biochemical alterations (Box 3), which all contribute to vascular endothelial dysfunction leading to vascular leakage and/or pathological angiogenesis (Antonetti et al., 2021; Duh et al., 2017). The endothelial master growth regulator VEGF modulates many aspects of vascular dysfunctions and thus plays central role in the pathogenesis and the treatment of DR. It is strongly upregulated in the vitreous of DR eyes and its neutralisation has been a game-changer in the treatment and management of DR. However, other extracellular factors play important roles as well (Antonetti et al., 2021). Notably, Gal-1 is also found significantly upregulated in the vitreous and aqueous humour of PDR patients (Abu El-Asrar and Ahmed, 2020; Kanda et al., 2017; Ridano et al., 2017). This is also reflected in mice modelling features of DR: chemically-induced diabetes leads to a significant and progressive increase of Gal-1 levels in retinal tissue (Kanda et al., 2017); both VEGF and Gal-1 are strongly upregulated in neovascular tufts in oxygen-induced retinopathy (OIR), a model of neovascular induction in response to relative hypoxia (Ridano et al., 2017). It is noteworthy, however, that this may merely be a general manifestation of the diabetic state, since at least in rats, the retina responds to diabetes induction with Gal-1 production (Bogdanov et al., 2014; Liu et al., 2009). Furthermore, in humans, Gal-1 was shown to be increased by 4.8 times in plasma samples obtained from type 2 diabetic patients (Liu et al., 2009).

Box 3 **Hyperglycaemia-induced biochemical changes.**

Hyperglycaemia has been proposed to damage cells by altering four major biochemical pathways. First, non-enzymatic glycosylation of proteins such as haemoglobin and basement membrane proteins leads to irreversible generation of advanced glycation end products (AGEs) in response to chronic hyperglycaemia (Brownlee, 2001; Duh, et al., 2017). In the retinal vasculature AGEs induce pericyte apoptosis and increased production of endothelial growth factors and inflammatory cytokines (Antonetti, et al., 2021). Second, high glucose concentrations also alter metabolic pathways, in particular that of polyol, with large expenses of NADPH, increases in oxidised glutathione and oxidative stress (Brownlee, 2001; Lorenzi, 2007). Third, increased flux through the hexosamine pathway (fructose-6-phosphate to UDP-GlcNAc) leads to increased modification of proteins by o-linked glycosylation. Fourth, hyperglycaemia also leads to *de novo* synthesis of diacylglycerol (DAG), an activator of protein kinase C (PKC) and the phosphorylation of its plethora of downstream effector molecules (Brownlee, 2001), many with important roles in vascular function (Geraldes and King, 2010). Indeed, inhibitors for different isoforms of PKC have been tested *in vitro* and *in vivo* with mixed results (Davis et al., 2009; Geraldes and King, 2010; Wu et al., 2018).
Activation of the renin-angiotensin pathway also plays an important role in establishing a DR phenotype in the retina (Wilkinson-Berka, 2006). Notably, many of these vascular and neuro-glial changes also lead to non-specific inflammatory and oxidative stress responses and the production and secretion of inflammatory mediators, such as IL-1β, IL-6, IL-8 and MCP-1, which have also been noted as potential biomarker of the disease (Youngblood et al., 2019).

Similarly, Gal-3 plasma levels are higher in patients with type 2 diabetes compared to controls (Jin et al., 2013). Both Gal-3 protein and its transcripts are also upregulated in serum and endothelial cells of mice with insulin-resistant diabetes (Darrow et al., 2011). Notably, the lack of Gal-3 aggravates this diabetic state and endothelial dysfunction, indicative of a positive correlation between levels of galectin-3 and insulin resistance, and of a protective role of Gal-3 in the pathogenesis of diabetes (Darrow and Shohet, 2015). In contrast, during chemically-induced diabetes, Gal-3(−/−) mice show significantly less retinal disease wt controls, in particular inner BRB dysfunction, junctional disruption and VEGF expression (Canning et al., 2007). Such specific functions of Gal-3 may reflect its role as a receptor for AGE and the relative importance of AGE-induced responses in the diabetic retina (Stitt, et al., 2005).

### Age-Related Macular Degeneration

AMD is arguably the most complex and multifactorial of retinal diseases. It is a leading cause of blindness in developed countries and associated with serious compromise of quality of life. Currently, at least half a million people suffer from advanced AMD, with numbers expected to climb in increasingly aged populations. Although the exact pathogenesis and aetiology of AMD are not well understood, the disease clearly affects choriocapillaris, Bruch’s membrane (the lamina between choroid and RPE), RPE and photoreceptors, and ultimately destroys the macula leading to loss of the high acuity vision (Fleckenstein et al., 2021). RPE dysfunction is at the heart of AMD pathogenesis, leading to photoreceptor death (from its apical side) and to atrophy of the choroid capillaries (from its basolateral side). Overall, AMD is categorised into two main clinical types. The slow-progressing atrophic (dry) form accounts for 85% of all cases and has no effective pharmaceutical treatment (Hadziahmetovic and Malek, 2021). Instead, cell-based therapy or RPE replacement treatments are being considered to treat dry AMD (Uyama et al., 2021; Vitillo et al., 2020). In its most severe form, dry AMD leads to complete atrophy of the RPE and the choroid and photoreceptors (geographic atrophy). Rapidly progressing neovascular (wet) AMD is a less common late manifestation of the disease and is seen for ca. 10% of all cases. It is characterised by pathological angiogenesis of the choroidal vasculature. The wet form of AMD is mainly VEGF driven and can be treated effectively with anti-angiogenics, especially anti-VEGF agents (Pugazhendhi et al., 2021).

Genetic non-modifiable risk factors for AMD have been identified. Ca. 35% of AMD patients have at-risk polymorphisms in the gene of complement factor H, suggesting that decreased innate immunity is linked with the disease (Fleckenstein et al., 2021). Another polymorphism associated with AMD is found in the ARMS2 gene, pointing to potential dysfunctional mitochondrial energy metabolism (Micklisch et al., 2017). In addition, life-style choices such as smoking, environmental factors and history of increased exposure to light are known to increase the risk of developing AMD (Heesterbeek and Thomas, 2020).

In a model of wet AMD, using laser-induced choroidal neovascularisation, Wu et al. show that Gal-1 is upregulated and required for lesion establishment, induction of VEGF receptor 2 signalling, as well as the upregulation of retinal EMT markers (Wu et al., 2019). Transcriptome analysis of human donor eyes comparing healthy and AMD retina and RPE/choroid samples identified many differentially expressed genes (Newman et al., 2012). In RPE/choroid samples, Gal-7, -2 and -8 were found upregulated in the macular region of patients with pre- or subclinical AMD, dry AMD, and geographic atrophy, respectively. In contrast, neuroretinal samples (devoid of RPE/choroid) showed Gal-8 and -12 downregulation in pre-AMD and Gal-3 upregulation in most forms of AMD. A quantitative proteomics study focusing only on the macular Bruch’s membrane/choroid of AMD patients revealed the upregulation of 56 (and downregulation of 43) proteins, with the majority linked to immune response functions (Yuan et al., 2010). Amongst these, Gal-3 was the most significantly elevated protein in samples of advanced dry AMD. In light of Gal-3 operating as a receptor for AGE in many settings, the authors then speculate on AGE involvement AMD. In addition to Gal-3, Gal-3 binding protein may also play a role, as it is secreted at 2–3 higher levels in RPE cells from AMD donors (An et al., 2006). Although few in number, these reports suggest that galectins may be critically involved in the pathogenesis of AMD, especially in aspects of the immune response.

### Other Retinopathies with Vascular Aetiology

Retinal vein occlusion (RVO), hypertensive retinopathy (HR) and retinopathy of prematurity (ROP) are conditions where retinal damage and visual impairment arise as a direct consequence of vascular dysfunction (Margalit and Srinivas, 2003).

RVO is caused by an obstruction of the central or branch retinal vein preventing drainage of blood from the inner retina. Bleeding and oedema are direct consequences of the blocked veins, and visual impairment occurs if the macula is involved. However, resultant ischaemia leads to neovascular complications (Blair and Czyz, 2020). Although galectin involvement in the pathogenesis of RVO has not yet been demonstrated, such a role is highly feasible given their demonstrated value as biomarker in ischemic cerebral stroke (Abel, et al., 2019). In addition, Gal-3 has been proposed as therapeutically protective in ischemic stroke and thus potentially in RVO (Wesley, et al., 2020).

HR is mainly caused by poorly controlled hypertension; however, factors such as smoking, genetic predisposition, gender and ethnicity can also play a predisposing role (Modi and Arsiwalla, 2020). Overall, the incidence of HR exceeds 60% among hypertensive patients. HR manifests as mild, moderate and severe retinopathy with symptoms ranging from general arteriolar narrowing and wall opacity leading to retinal haemorrhage, microaneurysm and hard exudates or even optic disc swelling (Erden and Bicakci, 2012; Tsukikawa and Stacey, 2020). Whilst galectins have not been directly implicated in the pathogenesis of HR, it is noteworthy that Gal-1, as a regulator of L-type CaV1.2 channels, may be an important regulator of blood pressure (Hu et al., 2018).

Retinopathy of prematurity (ROP) is a retinal vascular disease that was first described in 1940 as retrolental fibroplasia causing blinding in children (Dogra et al., 2017). The disease is a result of inappropriate dosage of oxygen administrated to preterm born babies. This leads to retinal vasoconstriction and endothelial cell death (Margalit and Srinivas 2003). Infants with severe forms of ROP may develop nystagmus (abnormal eye movements), bilateral retinal detachment and total blindness. ROP incidence is high in countries with low socioeconomic status, low access to health care facilities and well-trained ophthalmologists (e.g. India). ROP shares some pathogenic features seen with DR. Therapeutic efficacy of anti-VEGFs in treating and managing ROP underlines the importance of VEGF signalling (Tran et al., 2018) and also suggests a role for galectins.

### Genetically-Linked Retinal Degeneration

Lastly, there is a large number of hereditary retinal diseases that are characterised by progressive photoreceptor loss (Margalit and Srinivas, 2003). The most common inherited retinal disease is retinitis pigmentosa (RP), with more than 50 genes identified. RP is considered a rare disorder affecting about 1 in 4000. Most of the mutations that cause RP affect rod opsin (RHO), namely its protein synthesis and folding, activity, trafficking or recycling (in RPE), with many mutations mapped in RHO itself but also splicing factors or chaperones (Athanasiou et al., 2018; Athanasiou et al., 2013). Consequently, RP is characterised by rod dysfunction and underlying RPE atrophy, resulting in progressive loss of peripheral and night vision, often culminating in tunnel vision. Very rarely, there is also progression to defects in central high acuity vision.

Sustained chronic inflammation is likely to be an important element of RP pathogenesis and may explain why the disease can eventually also affect cones. Micro- and Müller glia are important regulators of the retinal inflammatory response (Karlen et al., 2020). Accordingly, in a mouse model of RP, Müller cells show features of gliosis and the production of pro-inflammatory cytokine tumour necrosis factor α (TNFα). Notably, Gal-3 expression is also elevated, presumably serving an immune-modulatory role (Roesch et al., 2012).

There is no treatment for RP. Food supplements such as vitamin A and E, DHA and lutein appear to delay retinal degeneration associated with RP. Given the genetic aetiology of RP, gene-therapy approaches are increasingly developed and trialled to directly repair the defective gene (McClements et al., 2020). In addition, transplantation may be used to replace degenerated retinal tissue (Uyama et al., 2021).

## Anti-angiogenic Agents to Treat Retinopathies

None of the described retinopathies can be cured. Treatments have been developed, which allow to manage, halt or in some instances revert some of the retinal damage and ensuing vision loss. As already mentioned above, clinical practice often involves surgical intervention and anti-inflammatory treatment. However, given the central role of vascular dysfunction and neovascularisation in PVR, ROP, DR and wet AMD, clinical care has aimed at halting microvascular damage for many decades. Until very recently, treatments with corticosteroids and laser photocoagulation were used to this effect, albeit with often significant side effects: cataract formation due to increased intraocular pressure for the former and visual field defects due to scarring and burns for the latter (Grosso and Panico, 2009). In the past 20 years, novel treatments have emerged based on the discovery of the central role of VEGF-A in retinal permeability and neovascularization (Ferrara et al., 2003). Anti-VEGF aptamers, antibodies and receptor fragments have been developed, which prevent activation of key VEGFR-mediated pathways, and in patients not only repair vessels and restore tissue morphology, but significantly also revert vision loss in many patients (Brown et al., 2013; Elman et al., 2012; Wells et al., 2015). Nevertheless, despite the benefits of even the best and most aggressive treatment of VEGF neutralising agents (with an average of eight injections per year), they are not at all times effective in the long duration: around half of all treated DMO patients have persistent macular oedema, do not respond with enhanced visual acuity, and need additional laser photocoagulation later on (Elman et al., 2012; Ford et al., 2013). What is more, anti-VEGF treatments are not usually used before the appearance of the vascular symptoms resulting in a lack of therapies for an earlier stage of retinopathies. Overall, these shortfalls in clinical efficacy have also highlighted the existence of other pathogenic, including additional angiogenic pathways, that may need to be targeted.

Aflibercept (marketed as Eylea®) (AFL) is the most recent and most effective of the currently clinically approved anti-VEGF drugs. It is routinely used in the UK and its NICE approvals include wet AMD and macular oedema (https://www.nice.org.uk). AFL is a pharmacologically engineered dimeric fusion protein consisting of the crystallizable fragment Fc portion of human Immunoglobulin Gamma1 (IgG1), which is fused to the ligand-binding domains of human VEGFR1 (D2) and VEGFR2 (D3). In contrast to other VEGF neutralising drugs based on monoclonal antibodies directed against VEGF-A, AFL has been designed to bind VEGFs at broader specificity and indeed shown to bind VEGF-A, VEGF-B and placental growth factors.

Clinical trials involving more than 2,400 people with DR showed that patients, who were treated with AFL, needed half as many injections over the course of one year than those treated with ranibizumab (Lucentis®), a Fab fragment of a high affinity anti-VEGF-A antibody, to reach similar endpoints in terms of vision preservation or recovery (Amoaku et al., 2020; Ross et al., 2020). Many studies collectively show that AFL, with its broader specificity, is clinically more effective.

Each AFL’s two polypeptide chains contains five putative N-glycosylation sites, all of which are occupied by glycans (https://www.drugbank.ca/drugs/DB08885). Indeed and similarly to the VEGF receptors, from which it is derived, AFL is heavily glycosylated (to approximately 15%), increasing its predicted amino acid molecular weight of 96.9 kDa to a total molecular weight of ca. 115 kDa. Gal-1, which binds to VEGF receptors, also binds to AFL with high affinity (K_D_ = 24 nM) (Kanda et al., 2015), and this may also explain its greater effectiveness in treating DR (see also below).

## Mechanisms of Galectin Function in Retinal Dysfunction

**FIGURE 5:**
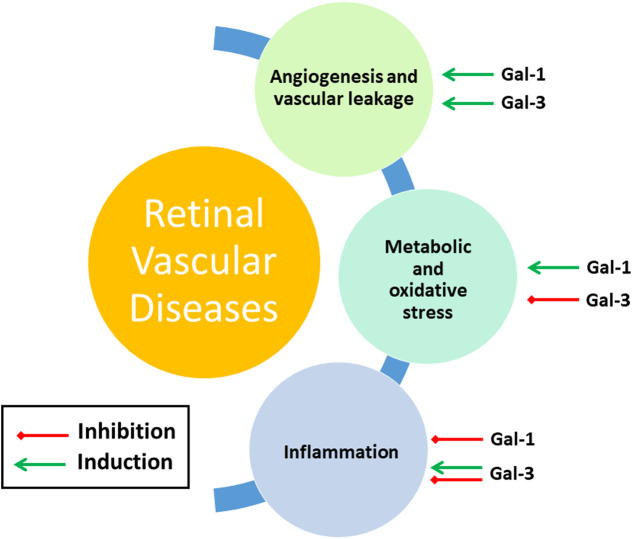
Gal-1 and Gal-3 involvement in main features of retinal vascular diseases. See Section *Mechanisms of Galectin Function in Retinal Dysfunction* for further details.

The above-described retinal disorders involve a number of recurring features, namely vascular and metabolic dysfunction, and inflammation. As discussed in the following, galectins, in particular Gal-1 and Gal-3, are intimately involved in regulating these processes, and this demonstrably also during retinal dysfunction (Figure 5).

### Angiogenesis and Vascular Leakage

Angiogenesis is the physiological formation of new vessels from pre-existing vessels (as opposed to de novo vasculogenesis) in response to a dynamic equilibrium between proangiogenic and anti-angiogenic factors. Tissue ischemia, hypoxia or inflammation generally tip the balance in favour of neovascularization. Sprouting angiogenesis, in which new vessels branch out via outgrowing tip cells, is by far the most frequent. Angiogenesis is a key pathogenic factor in several diseases and, in particular, in supporting solid cancer growths, which Judah Folkmann demonstrated to be angiogenesis-dependent, leading to decades of research into anti-angiogenic treatments for cancer (Carmeliet and Jain, 2011). Importantly, pathological angiogenesis is also a pathogenic hallmark of ROP, PDR, PVR and wet AMD (Bressler, 2009).

VEGF is considered the master regulator amongst the pro-angiogenic, paracrine and autocrine factors, which also include FGF, neuropilins, angiopoietins and TGF (Carmeliet and Jain, 2011). All three VEGF receptors (VEGFR1, 2, 3) are involved in the regulation of angiogenesis (Potente et al., 2011), but VEGFR2 is key in triggering the key prerequisite signals that induce endothelial cell proliferation, migration and polarization, in particular in response to VEGF-A. Angiogenic activation of VEGFR2 leads to the release and turnover of various second messengers such as phosphoinositides and Ca^2+^, and the activation of a plethora of protein phosphorylation cascades, including MAP and SRC family kinases (Moccia et al., 2019). The VEGF/VEGFR2 signalling axis is also critical in ocular angiogenesis, and this forms the mechanistic basis for the use of anti-VEGFs in many retinal disorders (Ferrara and Adamis, 2016).

Binding to and modulating growth factor receptors is a key function of many extracellular galectins, and there is abundant evidence that some galectins modulate the angiogenic process through interactions with VEGF receptors. Thus, these galectins may induce VEGF-independent, VEGF receptor-mediated angiogenesis, which may be involved in resistance to anti-angiogenesis drugs (Giuliano and Pagès, 2013; Yang et al., 2016).

Gal-1 is pro-angiogenic in the context of tumours, where it is frequently secreted by stromal fibroblasts (Zhu et al., 2016). There is increasing evidence that the pro-angiogenic activity of Gal-1 is achieved through co-opting VEGF receptor signalling both in retinal and non-retinal tissues (Kanda et al., 2015). Indeed, ocular neovascular induction is intimately linked to overexpression of Gal-1. Notably, PDR tissue shows elevated levels of Gal-1 (Kanda et al., 2015). In laser-induced CNV, neo-vessel formation, as well as angiogenic activities, are reduced in mice lacking the LGALS1 gene (Wu et al., 2019). In a mouse OIR model, retinal neovascularization and retinal hypoxia are significantly attenuated after Gal-1 inhibition by intravitreal injection of Gal-1-silencing adenoviruses (Yang et al., 2017). Similarly, pharmacological inhibition of Gal-1 by OTX008 suppresses retinal angiogenesis in the same model (Yang et al., 2017). Gal-1 expression in retinal endothelial cells has not been consistently shown. This may be due to loss of endothelial cells as a result of standard single cell isolation protocols. However, analysis by Lipski et al. show clear expression in endothelial cells (Lipski et al., 2020), suggesting that it may act in an autocrine fashion in endothelial cells. Among the non-vascular cells of the retina, Müller cells clearly express Gal-1 (Lukowski et al., 2019) and expression increases in response to interleukin (IL)-1β (Kanda et al., 2017) or oxidative stress *in vitro* (Abu El-Asrar and Ahmed, 2020). Interestingly, Gal-1 not only induces angiogenic responses in endothelial cells but also VEGF production in Müller cells, suggesting a feed-forward loop of both factors in the stimulation of endothelial VEGF receptors. In addition, there appear to be VEGF receptor-independent functions of Gal-1 that may modulate angiogenesis. For instance, its binding to endothelial CD146, which regulates angiogenesis and vessel leakage, induces apoptosis in human umbilical chord endothelial cells (Jouve et al., 2013).

Whilst there are no studies linking it to retinal angiogenesis, Gal-3 undoubtedly has angiogenic activity, in particular in a tumour environment (Dos Santos et al., 2017; Funasaka et al., 2014; Markowska et al., 2010). On the plasma membrane of endothelial cells, Gal-3 binds VEGFR2 and prevent its internalisation, with increased VEGFR2 phosphorylation and angiogenesis *in vitro* a direct consequence (Markowska et al., 2011). The same study also reports a significant reduction of suture-induced corneal neovascularization in Gal3(−/−) mice. Gal-3 is also involved in the angiogenic response of endothelial cells to the proteoglycan NG2, with which it forms a complex to induce α3β1 integrin signalling (Fukushi et al., 2004).

Similar studies show that Gal-8 induces endothelial cell migration and angiogenesis, possibly through interactions with CD166 (Delgado et al., 2011). In human umbilical chord endothelial cells and the chick chorioallantoic membrane, Gal-8 only activates cell proliferation and migration and angiogenic signalling in the presence of VEGF, suggesting synergistic signalling (Varinská et al., 2020). Gal-8 has also been shown to act specifically on human lymphatic endothelial cells and promotes lymphangiogenesis in a pathway involving integrin VEGF-C and podoplanin (Chen et al., 2016).

Neovessels formed by pathological angiogenesis are frequently leakier than their healthy equivalents. Indeed, angiogenesis and vascular leakage share many elements of the same signal transduction pathways. Whereas blood vessel leakiness in PDR and wet AMD is a consequence of the instability of the newly formed vasculature, it is the principal pathogenic manifestation of DMO, affecting the existing vasculature. Whilst endothelial permeability (often also referred to as basal vascular permeability) is the intrinsic property of the vascular wall to provide nutrients and molecular clearance for the underlying tissue, hyperpermeability, enabling excessive leakage of plasma protein and fluid from blood vessels to the interstitial space, is pathologic (Claesson-Welsh et al., 2020; Nagy et al., 2008). The vascular barrier is compromised in the event of hyperpermeability, and this is generally a result of reduced adhesiveness of paracellular junctions and morphological gap formation. However, different vascular beds display variances in the mechanisms underlying hyperpermeability. Particularly, in the brain and the retina, where paracellular contacts are made up of unique and highly impermeable junction complexes, changes in paracellular morphology are not as marked as in on-neural endothelium. Instead, leakage appears to occur also transcellularly via the induction of luminal caveolae, operating in tandem with the paracellular route (Turowski, 2017).

VEGF-A, originally known as vascular permeability factor, is among the extracellular factors that potently induce leakage via a dedicated receptor; others include histamine and bradykinin (Claesson-Welsh et al., 2020). All three have been shown to play a role in retinal vascular diseases (Klaassen et al., 2013; Urias et al., 2017). Additionally, angiopoietins also appear to play a role; however, they may affect leakage only indirectly. Importantly, all these leakage factors have been considered as targets for treating retinopathies, but only the neutralisation of VEGF has made it into the clinic thus far.

In the retina, VEGF-A is produced and released by various non-endothelial cell types, especially in response to hypoxia and inflammation (Nagy et al., 2007). Besides acting on ECs, it also regulates nonendothelial cells in the retina, such as retinal ganglion cells (Foxton et al., 2013; Park-Windhol and D’Amore, 2016). VEGFR2 is the main receptor to trigger endothelial permeability in response to VEGF-A (Dragoni, et al., 2021; Hudson, et al., 2014). Downstream activation of p38 MAPK, eNOS and SRC family kinases are crucial in mediating the paracellular response, including VE-cadherin phosphorylation and F-actin rearrangements (Koch et al., 2011; Orsenigo et al., 2012; Sun et al., 2012).

By virtue of binding and modulating surface growth factor receptor, galectins are also modulators of endothelial hyperpermeability. Indeed, two studies demonstrate a link between vascular hyperpermeability and Gal-1 and -8, respectively, in the tumour vasculature. Wu et al. found that Gal-1 expression correlates with elevated tumour vascular permeability in specimens of oral squamous cell carcinoma (Wu et al., 2014). In addition, these studies further show that Gal-1 increases vascular permeability in cultured endothelial cells by activating an NRP-1/VEGFR1 complex upstream of VE-cadherin and actin reorganisation. Moreover, tumour cells, which lack Gal-1, form smaller tumours with significantly less permeability *in situ* when transplanted into SCID mice. Similarly, Gal-8 also induces endothelial permeability *in vitro* and *in vivo* (Zamorano et al., 2019). It induces eNOS-mediated S-nitrosylation of p120-catenin and dissociation of adherens junction, both in venous endothelial cells and the mouse cremaster microcirculation. Thus, whilst in the retina there is clear evidence for a role of Gal-1 in pathological neovascularisation, a role in pathological retinal vessel leakage can only be inferred from anecdotal work in non-retinal models. Likewise, the involvement of Gal-3 and Gal-8 in retinal vascular leakage is likely but so far without direct evidence.

**FIGURE 6:**
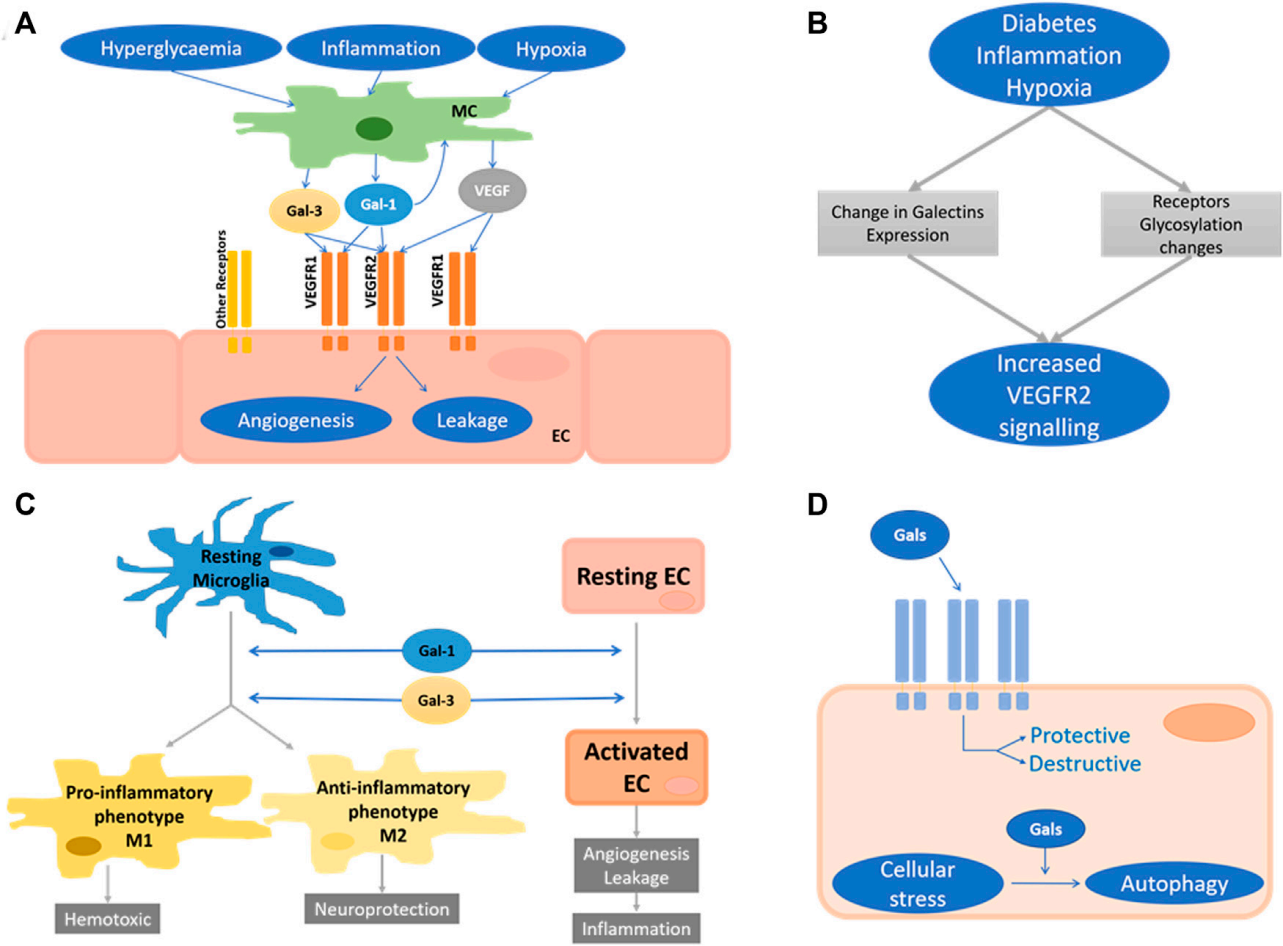
Unresolved areas in retinal galectins research. **(A)** Proposed roles of Gal-1 and Gal-3 in the regulation of angiogenesis and vascular leakage. Stimulated by hyperglycaemia, inflammation, and hypoxia, Müller cells (MC) secrete Gal-1, Gal-3 and VEGF-A, which activate VEGF receptors on endothelial cells (EC). Autocrine stimulation of EC may also occur. The molecular nature of the galectin-responsive VEGF receptors is still unclear. **(B)** Proposed interplay of galectin expression and receptor glycosylation in the activation of VEGF2 in response to pathogenic stimuli in the retina. **(C)** Proposed mechanism of the CNS/retinal galectins roles during neuroinflammatory response. Gal-1 and Gal-3 drive the microglia response toward both neurodegeneration and -protection. **(D)** Schematic of the intracellular and extracellular functions of galectins in any retinal cell type. They can be protective or disruptive.

In light of the criticality of both angiogenesis and vessel leakage in retinal disease, these processes should be studied in the context of galectins; in particular, in light of the success and the limitations of anti-VEGF treatment, pointing on the one hand to the clinical feasibility of anti-angiogenic treatment and, on the other hand to the existence of redundant and/or refractory pathways. Several key questions appear of importance, and these are summarised in Figure 6A. First, which of the retinal parenchymal cells produce galectins and in response to which pathological trigger? Clearly, Müller cells have been identified as the source of Gal-1 in response to oxidative stress and IL1, but autocrine regulation by endothelial cells is also supported by available expression data. In DR, hyperglycaemia could be an early, and inflammation a later trigger for Gal-1 secretion. Furthermore, given that in the diabetic state (i.e. hyperglycaemia), Gal-1 is upregulated and not all diabetics suffer from DR, the mere increase of Gal-1 may not be sufficient to induce angiogenesis or vascular leakage, in contrast to what confined laboratory data may suggest. Second, both Gal-1 and 3 can interact with the VEGF receptor, and any potential synergistic or antagonistic effect with each other and VEGF-A have yet to be explored. Crucially there is continued discrepancy in data as to exactly which VEGF receptor mediates Gal-1 (and Gal-3) function: both VEGFR1 and R2 have been shown to be modulated by Gal-1 (Croci et al., 2014; Markowska et al., 2011; Wu et al., 2014). Gal-1 and Gal-3 individually activate VEGFR2 (D’Haene et al., 2013; Markowska et al., 2011), whereas the combination of these two galectins can lead to VEGFR1 activation (D’Haene et al., 2013) (Figure 6A). Whilst these discrepancies undoubtedly reflect the differences of the endothelial cells and vasculature beds that had been investigated, this also indicates that established molecular principles for galectins cannot be directly transferred to retinal disorders.

### Changes in Proteoglycan Expression

Galectins display binding affinity for β-galactosides found in both N- and O-linked glycans. Both of these post-translational protein patterning systems are substantially influenced by pathology. Therefore, the galectin response is not only differentially regulated by process-specific galectin expression but crucially also by changes in the cellular complement of proteoglycans. Notably, as recently reviewed by Reily et al., the glycosylation patterns of a wide variety of transmembrane receptors differ in response to varying physiological and pathophysiological stimuli, including during inflammation, cellular stress, and vascular dysfunction (Reily et al., 2019). For instance, the binding specificity of CD43 and CD45 on B and T cells is heavily dependent on O- and N-linked glycosylation and this also regulates the binding affinity of Gal-1, at least to CD45. Indeed, glycosylation plays an important role in the innate and adaptive nature of all immune and inflammatory responses. In the brain, recent research shows that amyloid β25-35, a neuroinflammatory peptide, induces changes in O-glycosylation and that this contributes to neuroinflammation in the hippocampus of rats (Ramos-Martinez et al., 2018). Phagocytosis of dysfunctional neurons by activated microglia depends on desialylation of cell surface glycans and the creation of binding for Gal-3, which then acts as an opsonizing factor in preparation for subsequent endocytosis (Nomura et al., 2017). Similarly, Gal-8-glycan binding activity is significantly reduced by desialylation in endolysosomal damage response, immunosuppression, and neuroprotection (Pardo et al., 2019; Pardo et al., 2017).

Metabolic changes such as hyperglycaemia have long been shown to lead to glycan changes, especially in O-linked glycosylation (Reily et al., 2019). Altered glycosylation is also a universally recognised change in cancer, with some glycans used as biomarkers of disease progression. Of interest for galectin biology is the altered branching of N-glycans. Cell transformation is generally associated with increased GlcNAc transferase-V activity, which catalyses N-glycan branching; this in turn frequently results in increased addition of N-acetyl-lactosamine and higher galectin binding activity (Bousseau et al., 2018). Pro- and anti-inflammatory stimuli lead to remodelling of endothelial cell glycosylation, with anti-inflammatory stimuli and hypoxia inducing a Gal-1-permissive phenotype (Croci et al., 2014). These glycosylation changes strongly affect VEGFR2 and allow signalling in the absence of VEGF, mimicked by Gal-1. Whilst, in relation to cancer biology and therapy, these findings explain a mechanism of VEGF-refractory tumour angiogenesis (Croci et al., 2014; Very et al., 2017), they also point to plasticity of endothelial cells and their responsiveness to growth factors in angiogenesis and possibly other key endothelial cell functions (Figure 6B). Similarly, Gal-3 interacts with glycans on extracellular matrix glycoproteins such as VEGFR2, CD43, CD45, vitronectin and integrin 1, thus affecting angiogenesis, cellular adhesion, and apoptosis in cancer (Johannes et al., 2018).

Indeed, the regulation of angiogenesis, including endothelial proliferation and migration by glycosylation, has been extensively studied and is reviewed elsewhere (Bousseau et al., 2018). Unsurprisingly, whilst not yet extensively studied, regulatory mechanisms based on altered proteoglycan expression are also thought to play a pivotal role in ocular angiogenesis (Markowska et al., 2014). In the retina, Gurel et al. show that O-GlcNAc patterns change during postnatal retinal vascular development and neovascularization, and their dysregulation during hyperglycaemia may contribute to the pathogenesis of DR (Gurel et al., 2013). Hyperglycaemia increases flux through the hexosamine biosynthetic pathway, which then directly feeds into the biosynthesis of O-linked GlcNAc glycans and may thus affect the pathogenesis of microvascular dysfunction, including that seen in DR (Semba et al., 2014). Analysis of proteoglycan expression in tear fluid revealed unremarkable differences between healthy controls and individuals from three diabetic disease groups, indicating that, at least in tears, diabetes-related ocular glycan changes are not detectable (Nguyen-Khuong et al., 2014). In contrast, Inafuku et al. identified changes in glycophenotype, namely Gal-1-favouring N-glycans, in the vitreous humour of human PDR retinae (Inafuku et al., 2015). In addition, Ridano et al. show that in OIR glycosylation shifts toward a pattern permissive for Gal-1 binding, and this particularly in areas of neovascularization (Ridano et al., 2017), suggesting that findings from tumour angiogenesis (Croci et al., 2014) are indeed transferable to retinal pathologies. It remains to be shown if these changes are associated with altered galectin binding to and activation of any retinal cell. Future studies should also establish if glycosylation changes can induce Gal-1-mediated VEGFR2 activation in the absence of VEGF-A and thus contribute to VEGF-refractory retinal disease. Analogous to recent advances in tumour research, retinal tissue from patients or relevant animal disease models, but also cell culture models should be systematically interrogated with lectin panels or, better still, subjected to mass spectrometry-based glycome analyses.

### Inflammation

Neuroinflammation is an early sign of a wide variety of CNS and PNS disorders, including those of the retina (e. g. AMD, RP, DR). Compared to the periphery, the neuroinflammatory response in nervous systems is slower and weaker and mainly controlled by the activity of glial cells, in particular microglia, astrocytes (and in the retina Müller glia). Neurons, endothelial, resident parenchymal cells and cells migrating from the peripheral blood (e.g. monocytes and lymphocytes) also play an important role in orchestrating the synchronized action of pro- and anti-inflammatory processes, including the production and release of cytokines, chemokines and reactive oxygen species (Fonseca et al., 2014; Ransohoff et al., 2015). Microglia have inflammatory functions analogous to those of macrophages, and their activation generally leads to increased levels of pro-inflammatory cytokines, such as IL-1β, IL-6, IL-8 and TNF-α. During retinal disease, microglia and retinal astrocytes are chronically activated and secrete IL-6, MCP-1 and VEGF. In diabetes, Müller glia present with enhanced levels of AGE receptors and enhanced production of cytokines, including TNF-α, IL-1β, IL-6, MCP1, nitric oxide and VEGF (Rübsam et al., 2018; Shin et al., 2014; Xu et al., 2018). Indeed, during retinopathy progression, glial cells communicate with each other and with neurons via secreted factors to induce a pathological state characterised by inflammation, oxidative stress and neurodegeneration (Karlstetter et al., 2015; Vecino,et al., 2016). Understanding the balance between pro- and anti-inflammatory processes that maintain retinal homeostasis is important to define pathogenesis and identify novel therapeutic targets (Dick, 2017). Roles in regulating both pro- and anti-inflammatory processes have been described for the galectins expressed in the CNS and retina.

Gal-1 tempers microglia activation in the context of inflammation-mediated neurodegeneration (Starossom et al., 2012). In mice *in vivo*, the absence of Gal-1 leads to more severe experimental autoimmune encephalomyelitis and is associated with more inflammatory microglia and more severe demyelination. Gal-1 also inhibits LPS-induced microglial activation *in vitro* and, when used therapeutically in mice, prevents degeneration of dopaminergic neurons, presumably by modulating microglial MAPK/IκB/NFκB signalling (Li et al., 2020). Moreover, Gal-1 is also neuroprotective by inducing proliferative and antioxidant activities in support of myelination and neurogenesis (Siew and Chern, 2018; Bonsack and Sukumari-Ramesh, 2019). In the retina, Müller cells and astrocytes express and secrete Gal-1. Whilst potential anti-inflammatory or neuroprotective roles of Gal-1 have not specifically been investigated in the context of retinal disorders, it has been reported that in zebrafish photoreceptor death in the zebrafish retina induces the expression of a galectin-1-like protein (Drgal1-L2) in microglia and proliferating Müller cells (Craig et al., 2010). Notably, knockdown of Drgal1-L2 results in reduced regeneration of rod photoreceptors, supporting a role in regenerative neurogenesis.

Gal-3 is generally upregulated in neuroinflammatory disorders, where it can promote both pro- and anti-inflammatory immune functions. Its expression is also upregulated in multiple sclerosis and a lack of Gal-3 is associated with less severe autoimmune encephalomyelitis (Jiang et al., 2009). Similarly, Gal-3 appears not to be expressed in the neuroretina of healthy mice but upregulation is seen during EAU in the retinal endothelium (Lipski et al., 2020). Nevertheless, a potential Gal-3 pathogenic function in EAU has yet to be demonstrated. During the ischemic immune response in the brain, Gal-3 appears to act at various points, potentially with a more proinflammatory role during early, and a protective role during the late degenerative pathology (Rahimian et al., 2018; Siew and Chern, 2018; Thomas and Pasquini, 2018). Similarly, in traumatic brain injury, both neuroprotective and neurodegenerative immune roles for Gal-3 have been described, and these may differ depending on the severity and stage of progression of the injury (Nishikawa and Suzuki, 2018; Siew and Chern, 2018). Recently, supporting a Gal-3 pro-inflammatory role in the retina, Mendoca et al. showed that diabetes-induced neuroinflammation of the optic nerve tissue is strongly reduced in Gal-3 −/− mice, manifested by a reduction in iNOS and GFAP and improved axon myelination (Mendonça et al., 2018).

Gal-4 plays a prominent role in the regulation of myelination pattering of nerve axons (Siew and Chern, 2018). However, also given that myelination of nerves stops in the retina, no role or cell expression for Gal-4 in retinal biology has so far been found (Lipski et al., 2020).

Gal-8 is neuroprotective and antibodies blocking its functions are found in autoimmune and inflammatory disorders. Accordingly, it protects hippocampal neurons *in vitro*. Engineered lack of LGALS8 in mice leads to more severe experimental autoimmune encephalitis, presumably by shifting the T cell balance to a Th17 phenotype (Pardo et al., 2017). Similarly to Gal-3, Gal-9 also appears to serve several roles in the immune response of nervous systems, namely at the astrocyte-microglia interface (Steelman et al., 2013; Steelman and Li, 2014), the microglia response during multiple sclerosis (Stancic et al., 2011), and toxoplasmic encephalitis in mice (Liu et al., 2018). In contrast, studies by Liang T et al. suggest that Gal-9 could promote recovery during intracerebral haemorrhage-induced injury potentially by its binding of Toll-like receptor-4 (Liang et al., 2020).

Collectively these studies indicate that many of the CNS/retinal galectins may have differential roles during the course of the neuroinflammatory response. Crucially, Gal-1 and Gal-3, both with demonstrated pathogenic roles in the retina, have the potential to push the microglia response toward neurodegeneration and -protection. In the context of retinopathies, this calls for well-executed studies in which the temporal aspects and differential, multistep activities of microglia are fully taken into account (Figure 6C). Given that neutralisation of both Gal-1 and Gal-3 holds potential as targets to normalise vascular dysfunction (see above), it is crucial that their overall pathogenetic role is fully reconciled with regard to potential pro- and anti-inflammatory functions. Notably, this is analogous to VEGF, for which neutralising therapies demonstrate clear benefits for the vascular pathology (Duh et al., 2017) but may impair neuronal function (Foxton et al., 2013; Park-Windhol and D’Amore, 2016).

### Metabolic and Oxidative Stress

A diverse range of disorders are associated with a pronounced level of oxidative stress and amongst these, many of the retinopathies. One of the reasons is the chronic overactivation of the inflammatory retinal response to external damaging stimuli, which is responsible for tissue destruction and remodelling underlying irreversible retinal pathologies (e.g. AMD, DR or retinal detachment). The retina, which is one of the highest oxygen-consuming tissues in the body, is particularly sensitive to enhanced ROS levels associated with oxidative damage of organelles and molecules. Indeed, antioxidants have shown to provide beneficial effects on the development of retinopathies in animal studies, but similar benefit in patients has yet to be demonstrated (Akhtar-Schäfer et al., 2018; Kowluru and Chan, 2007).

Oxidative stress in the retina leads to endothelial dysfunction, which directly leads to the pathological vascular phenotype seen e.g. in many patients with diabetes. Mitochondria, enzyme systems and photosensitizers in the endothelial layer produce the rise in ROS caused by the overproduction of superoxide. In addition, different studies show worsening hyperglycaemia correlates with non-enzymatic glycosylation of the endothelium, high levels of ROS and AGEs and the following endothelial dysfunction (Nowotny et al., 2015). Similarly, high ROS levels and the resultant oxidative damage are also correlated to retinal ageing (Beatty et al., 2000). In addition, much of the retinal ROS accumulates in the RPE with its high metabolic rates of spent retinal constituents but also light absorption, and any excess of ROS production disturbs redox homeostasis and leads to oxidative stress. Mitochondria, as the major source of ROS, incur damage and dysfunction (Nita and Grzybowski, 2020; Ruan et al., 2021). Indeed, many studies have shown a link between mitochondrial oxidative damage in the RPE and photoreceptor dysfunction in patients with AMD (Abokyi et al., 2020).

Gal-1 is directly linked to AGE accumulation in the retina. Gal-1 accumulation and subsequent vascular dysfunction in the diabetic retina appears to be directly linked to the exposure of microglia and macrophages to raised AGE (Kanda et al., 2017). Conversely, as a bona fide AGE receptor, Gal-3 is clearly protective against oxidative damage (Abel et al., 2019). Raised endothelial dysfunction and clotting predispositions are observed in Gal-3-KO diabetic mice compared to wt (Darrow et al., 2011). Genetic deletion of Gal-3 also aggravates hyperglycaemia through the downregulation of glucose transporter type 4 (glut-4) in the endothelium (Darrow et al., 2011). Lastly, Canning et al. demonstrated that Gal-3 acts as an AGE receptor in the retina and that diabetes in Gal-3 KO mice leads to overall less severe retinal disease, and specifically milder BRB dysfunction and reduced retinal VEGF levels (Canning et al., 2007).

Oxidative damage of intracellular proteins and organelles also requires autophagy for cellular clearance and homeostasis (Filomeni et al., 2015). Dysregulated autophagy is associated with many retinal disorders, including DR, AMD and RP, and its promotion may be associated with improved clinical outcome (Moreno et al., 2018). Upon autophagy initiation, cytoplasmic cargo is engulfed by double-membrane vesicles that develop into elongated and mature autophagosomes. The latter is then fused with lysosomes, where the intracellular content is degraded by lysosomal peptidases and hydrolases (Koepke et al., 2020). Interestingly, galectins are found to accumulate quickly around different endocytic vesicles and compartments upon disruption. Notably, Gal-8 interacts via its C-terminus with the autophagic receptor NDP52 (Kwon and Song, 2018), and Gal-3 was shown to inhibit Gal-8 driven autophagy (Cheng et al., 2017). Moreover, tripartite motif-containing (TRIM) proteins, another type of autophagy receptors, interact with galectins (Chauhan et al., 2016). Thus galectins may play roles at various stages during cellular cargo degradation (Johannes et al., 2018).

Collectively, and in analogy to the discussion relating to neuroinflammatory functions of galectins, the neutralisation of galectins may be counterproductive to beneficial clinical outcomes in retinal disorders as this may negatively impact on how the diseased tissue copes with metabolic and oxidative stress, including organelle damage. Thus, the role of galectins needs to be investigated holistically, taking into account all retinal cell types and various stages of disease. Studies should also focus on the location of galectin function since their intracellular and extracellular function in the retina may possibly be segregated into disease attenuating and disease-promoting activities (Figure 6D).

### Clinical Translation

The eye has long been recognised as being at the forefront of therapeutic research. It is an easily accessible organ, many physiological and pathophysiological features of which can be monitored by ever increasingly sophisticated, non-invasive imaging. Importantly, it is also immunologically privileged and overall not affected by many of the complications encountered with systemic drug delivery; indeed, most ocular therapeutics are delivered locally, with mostly insignificant systemic accumulation. As such, the eye has been at the centre of pioneering treatments involving corrective gene and stem cell therapies (MacLaren et al., 2016). Whilst these ground-breaking therapies have started to change patients’ lives in extraordinary fashion, insights gained will undoubtedly benefit the development of such advanced therapeutic approaches in other organs.

Pathological neovascularization in the retina could be targeted by Gal-1 antagonists. In light of the above discussed pre-clinical mechanistic and circumstantial clinical evidence positing Gal-1 as a VEGF-independent activator of VEGFR2 in retinal endothelial cells, it may well be a driver of anti-VEGF refractory disease. Whilst there is currently no direct clinical evidence that Gal-1 inhibition could be beneficial for patients with DR, there are indirect indications: as mentioned above, AFL has clinically been shown to be superior to other anti-VEGF agents. This may be due to its potential to bind and neutralise VEGF family members other than VEGF-A; however, it remains to be demonstrated that other VEGF family members play a significant role in diabetes-induced vascular dysfunction. For instance, permeability in neural endothelium is triggered by VEGF-A but not PlGF-1 (Hudson et al., 2014). Notably, AFL also binds Gal-1 (Kanda et al., 2015), and physiological AFL levels precipitate Gal-1 *in vitro* (Waltl et al., 2018). Together with increasing evidence for a pathological role of Gal-1 in DR (Abu El-Asrar and Ahmed 2020), these findings suggest that enhanced therapeutic efficacy of AFL in retinal diseases may be due to its additional effect on Gal-1. Future studies should determine if therapeutic targeting of Gal-1 in combination with a pure VEGF-A anti-angiogenic delivers the superiority seen with AFL.

A number of compounds have been developed to target galectins specifically, many of which are at an advanced stage of development and already used in clinical trials with a particular focus on cancer and fibrotic disease (Table 2). To target galectins, the first port of call is to exploit their glycan-binding functions. However, pure glycans make for poor galectin antagonists (Bertuzzi et al., 2020). Firstly, galectin-glycan only interactions are typically relatively weak (µM-mM range). Secondly, they are also highly promiscuous and not very selective for individual galectin family members. Lastly, from a pharmacokinetic point, glycans make for poor therapeutic drugs due to their high hydrophilicity and, consequently, low cell penetration. In addition, their metabolic instability precludes them from being feasible drugs (Ernst and Magnani, 2009; Hevey, 2019). Instead, structural and functional glycomimetics with superior pharmacokinetics have emerged as molecules of choice. However, whilst they are metabolically more stable and can be designed for improved tissue and cell penetration, they are still primarily designed to target the highly conserved CRD and thus rarely display specificity for a single galectin. Galectin-targeting glycomimetics are often divided into monovalent and multivalent (lattice-like) molecules, which are either based or not on carbohydrate chemistry (Bertuzzi et al., 2020).

**TABLE 2:** Compounds targeting galectins in clinical trials.

	Compound	Clinical Trial/NCT
Gal-3	TD139	Idiopathic Pulmonary Fibrosis (NCT03832946)
		COVID-19 (NCT04473053)
	GCS-100	Chronic Kidney Disease (NCT01843790)
		Chronic Lymphocytic Leukemia (NCT00514696)
		Multiple Myeloma (NCT00609817)
	Belapectin	Varying Degrees of Hepatic Impairment (NCT04332432)
Gal-1/3	GM-CT-01	Melanoma (NCT01723813)
		Metastatic Colorectal Cancer (NCT00110721)
		Solid Tumors (NCT00054977)
Gal-1	OTX008	Advanced Solid Tumors (NCT01724320)

Amongst a large group of related monovalent carbohydrate-based molecules, TD139 stands out as possibly the most clinically advanced galectin-antagonist. It is mainly used to target human Gal-3 and is being trialled in treating idiopathic pulmonary fibrosis (NCT03832946) (Hirani et al., 2020) and COVID-19 (NCT04473053). Other clinically explored carbohydrate-based molecules comprise GCS-100 and belapectin, both Gal-3-selective multivalent polysaccharides derived from citrus pectin, and GM-CT-01, a modified galactomannan oligomer that binds both Gal-1 and Gal-3 (Table 2). More recent efforts in the development of carbohydrate-based galectin inhibitors focus on chemical modifications that increase the selectivity for disease-specific galectin-glycoprotein complex (Bertuzzi, et al., 2020).

Anginex, a non-carbohydrate glycomimetic galectin inhibitor, is a designed amphiphatic β-sheet peptide, which preferentially targets Gal-1 and has anti-tumour and -angiogenesis properties. Many improved molecules have been derived from it and characterised pre-clinically (Wang et al., 2012). OTX008 is a chemically very distinct calixarene derivative designed to topologically mimick anginex, and it has emerged as arguably the clinically most promising Gal-1 antagonist to date. Preclinically it has shown tumour growth inhibition in models of hepatocellular (Leung et al., 2019) and head and neck squamous carcinoma (Koonce et al., 2017), but also glioblastoma and ovarian cancer (Zucchetti et al., 2013).

Given that galectins fulfill a plethora of functions, their systemic targeting is likely to be associated with complex side effects. Not only could pre-clinical (and potentially clinical) targeting of galectin in retinal disease offer therapeutic benefits, but also opportunities toward a better understanding of their pharmacological usefulness in a well-defined, mostly isolated organ. For instance, galectin-targeting in pre-clinical models of retinal disease could provide a better understanding of the seemingly opposing functions of Gal-1 and Gal-3 in the regulation of microglia and vasculature of nervous systems.

## Conclusion and Future Perspectives

Collectively, diseases of the retina are widespread and lead to a growing number of individuals with vision impairment or blindness, both in the elderly and working-age population. Basic and clinical research has advanced at pace to understand the underlying complex pathologies and find an effective treatment. This is clearly reflected in related scientific output (as listed in PubMed), which grew exponentially since 2001, surpassing 10,000 publication in 2020. Within this area, focus on galectins also gained some traction with increasing numbers of publications since around 2000. However, after peaking at around 10 publications in 2017, the number of outputs has been decreasing. Given the clear links of galectins to retinal disease discussed above, this suggests that this area of research warrants stimulation as many important questions remains to be explored.

In particular, evidence of galectin involvement in cancer angiogenesis predisposes members of this versatile lectin family for an essential role in retinal vascular dysfunction. Furthermore, their involvement in the regulation of microglial activity in the brain also suggests a similar role in the retinal immune modulation, the targeting of which holds great potential. Clinically, galectins could be part of the answer in explaining retinal vascular disease refractory to anti-VEGFs, as strongly suggested by similar observation in tumour angiogenesis. The multifunctional nature of galectins certainly fits that of complex diseases such as DR and AMD. Lastly, we also strongly believe that basic galectin research will benefit from focused research in the eye. Not only has the eye become key in understanding basic aspects of angiogenesis and vascular leakage, but also have novel technologies made it the most accessible of organs for clinical and experimental non-invasive *in vivo* imaging. Thus, many observations made in the retina can serve to understand similar processes, in particular for complex interactions between the neurovascular, immune and neuronal cells.

## Abbreviations

AFL, aflibercept; AGE, advanced glycation end product; AMD, Age-related macular degeneration; BRB, blood-retinal barrier; CRD, carbohydrate recognition domain; DMO, diabetic macular oedema; DR, diabetic retinopathy; EAU, experimental autoimmune uveitis; EMT, epithelial to mesenchymal transition; HR, hypertensive retinopathy; IL, interleukin; NPDR, nonproliferative DR; OIR, oxygen-induced retinopathy; PDR, proliferative DR; PKC, protein kinase C; PVR, proliferative vitreoretinopathy; RHO, rod opsin; ROP, retinopathy of prematurity; RP, retinitis pigmentosa; RPE, retinal pigment epithelium; RVO, retinal vein occlusion; VEGF, vascular endothelial growth factor; VEGFR, VEGF receptor.
